# Disease outcomes following lateral switch among different CD20-antibodies in active multiple sclerosis

**DOI:** 10.1177/13524585251361330

**Published:** 2025-07-30

**Authors:** Franziska Axhausen, Anne Mrochen, Pia Winter, Romy Baumgart, Pauline Mühlenbrock, Anna Mück, Stephanie Wolff, Sven G Meuth, Kathrin Möllenhoff, Steffen Pfeuffer

**Affiliations:** Department of Neurology, University Hospital Giessen and Marburg, Justus-Liebig-University Giessen, Giessen, Germany; Department of Neurology, University Hospital Giessen and Marburg, Justus-Liebig-University Giessen, Giessen, Germany; Department of Neurology, University Hospital Giessen and Marburg, Justus-Liebig-University Giessen, Giessen, Germany; Department of Neurology, University Hospital Giessen and Marburg, Justus-Liebig-University Giessen, Giessen, Germany; Department of Neurology, University Hospital Giessen and Marburg, Justus-Liebig-University Giessen, Giessen, Germany; Department of Neurology, University Hospital Giessen and Marburg, Justus-Liebig-University Giessen, Giessen, Germany; Department of Neurology, University Hospital Giessen and Marburg, Justus-Liebig-University Giessen, Giessen, Germany; Department of Neurology, University Hospital Duesseldorf, Heinrich-Heine-University Duesseldorf, Dusseldorf, Germany; Institute of Medical Statistics and Computational Biology, Faculty of Medicine, University of Cologne, Cologne, Germany; Department of Neurology, University Hospital Giessen and Marburg, Justus-Liebig-University Giessen, Giessen, Germany

**Keywords:** Multiple sclerosis, ofatumumab, ocrelizumab, hypogammaglobulinemia, lateral switch

## Abstract

**Background:**

Ocrelizumab (OCR) and ofatumumab (OFA)are approved and their differences in dosing route and interval allow personalized treatment. However, there are no data on whether lateral switches between both substances affect treatment effectiveness or safety.

**Methods:**

We screened our local cohort of MS patients, who began OCR since 09/2020 or OFA since 09/2021. Patients with a lateral switch were matched to controls who continuously received initial B-cell depleting therapy (BCT). We compared disease courses including effectiveness outcomes as well as peripheral CD19+ B-cell counts and serum IgG levels.

**Results:**

From 09/2020 to 03/2024, 713 patients were subjected to BCT (OCR: 396; OFA: 317 [as in Fig.1]). The matched OCR cohort included 38 switchers and 149 controls. The OFA cohort consisted of 24 switchers and 83 controls. Effectiveness outcomes were comparable among switchers and controls. B cell depletion appeared slightly pronounced following a switch. Serum IgG levels declined faster among switchers compared to controls (OCR: 9.7 vs 9.0 g/L; *p* = *0.007*; manifest hypogammaglobulinemia (HGG) in 13.2% vs 6.0%; OFA: 9.7 vs 8.4 g/L; *p* = *0.016*; manifest HGG in 8.3% vs 2.4%).

**Conclusions:**

Lateral switching between BCT does not abate effectiveness in this matched real-world cohort. Our observation of increased loss of IgG warrants further validation, but may indicate niche-specific immunological effects of OFA and OCR.

## Introduction

Anti-CD20 therapies are well-established disease-modifying therapies (DMT) for active relapsing multiple sclerosis (RMS). Ocrelizumab (OCR) includes administration of two intravenous doses of 300 mg each within 2 weeks and maintenance treatment of 600 mg intravenous semiannually.^1^ Subcutaneous ofatumumab (OFA) was approved for monthly application (20 mg subcutaneous).^2^

OCR and OFA demonstrated superior efficacy in phase 3 clinical trials, showing suppression of inflammatory disease activity and delayed disability worsening.^1,2^ Their effectiveness was deemed comparable in real-world studies.^3–5^

Although current data showed generally favorable safety and tolerability, long-term side effects have emerged among patients receiving anti-CD20 therapy.^5,6^ Most prominent is the reduction of serum immunoglobulin (IgG) levels, which can ultimately result in manifest hypogammaglobulinemia (HGG) and associated infections.^7–9^

The evidence regarding OFA-associated HGG is inconsistent: while core and extension studies reported no substantial IgG decline,^5^ real-world data indicated a similar reduction of IgG levels in patients treated with OFA compared to patients treated with OCR.^10^ Risk factors for HGG include age, a higher number of previous therapies and a longer duration of anti-CD20 therapy.^7–9^ Whereas data from long-term extension studies showed no clear association of HGG and severe infections,^5,6^ real-world data suggested that HGG is associated with a higher risk of symptomatic or serious infections.^7^

Continued exposure to B-cell depleting therapy (BCT) appears to be the strongest risk factor for IgG reduction and development of HGG.^8^ Yet, little is known about whether switching between different BCTs further increases this risk.

Thus, we evaluated the disease outcomes, as well as peripheral B cell counts and serum IgG levels in two independent, propensity-matched, prospective cohorts of OCR and OFA patients with and without lateral BCT switch.

## Methods

### Patients

Patients, subjected to receive either OCR or OFA for treatment of active RMS according to 2017 revised McDonald criteria,^11^ were enrolled into our local prospective registry. Treatment decisions were made independently of registry inclusion, based on national and international guidelines, and the most recent summary of product characteristics.

Within our registry, prospectively collected parameters included baseline demographics, relapses, magnetic resonance imaging (MRI) parameters, expanded disability status scale (EDSS) scores as well as serum immunoglobulin G (IgG) levels and peripheral CD19+ B-cell counts. Furthermore, OCR infusions were documented, and OFA injections were regularly assessed to ensure drug adherence.

For the current study, we evaluated all patients subjected to OCR or OFA. In these two independent cohorts, we excluded all patients who underwent extended interval dosing,^12^ received previous BCT or immune reconstitution therapies (alemtuzumab/cladribine), and patients with progressive forms of MS. Among the remaining patients, we compared those who underwent a lateral switch between OCR and OFA with those patients who remained on the respective treatment following propensity score-matching (PSM).

### Treatment outcomes

Patients were evaluated semiannually during clinical routine as well as on unscheduled visits following clinical relapses. Assessments were conducted as previously published.^4,13^

We used a composite endpoint combining clinical relapses and new or enlarging T2L summarized as “inflammatory disease activity” (IDA). During clinical routine peripheral CD19+ B-cell levels were measured using flow cytometry and serum IgG levels were assessed. All tests were conducted at our hospital in the accredited laboratory.

### Statistical analysis

We separated all patients into two cohorts depending on the first CD20-antibody administered. Within these both “cohorts” (“OCR cohort” and “OFA cohort”), we performed PSM to select suitable controls for the “switch group” within. For each subject, a propensity score was calculated using a logistic regression model using “treatment switch” as dependent variable and “age,” ‘sex “MS duration since onset,” ‘annualized relapse rate at baseline “EDSS at baseline,” “number of T2L at baseline,” “number of previous DMT” and “last previous DMT” as covariables.

Matching was performed based on the propensity scores using the nearest neighbor matching without replacement. We allowed a variable ratio of up to 5:1 and a caliper of 0.1 standard deviations (SD) of the propensity score^14^ similar to previous studies comparing different BCT.^15^ Covariate balance was assessed by standardized mean difference (SMD), where a difference of less than 0.1 was considered as an acceptable balance.

Further analyses were performed entirely on the matched “cohorts” whereas we analyzed the OCR-“cohort” consisting of the “groups”: “OCR-switch” vs “OCR-control” and the OFA-“cohort” consisting of the “groups”: “OFA-switch” vs “OFA-control,” separately. We did not compare OCR and OFA subgroups to each other.

For evaluation of effectiveness, we generated Kaplan-Meier plots using IDA and confirmed worsening of disability (CDW) as outcome parameters. Significance levels were calculated using a log-rank test. Further analyses were performed using descriptive statistics including calculation of annualized relapse rates (ARR) and incidence rates per 100 patient-years (IR). Continuous data were compared using an unpaired *t* test. A *p* value below 0.05 was considered significant. All analyses were performed using R (R Core Team, 2024, version 4.4.1).

### Ethical approval

All patients gave written informed consent. The study was approved by the local ethical committee (Ethical board of the Medical Faculty of the Justus-Liebig-University Giessen; 53/23).

### Data availability

Anonymized patient data will be shared upon reasonable requests from qualified investigators.

## Results

### OCR cohort

**Figure 1.**
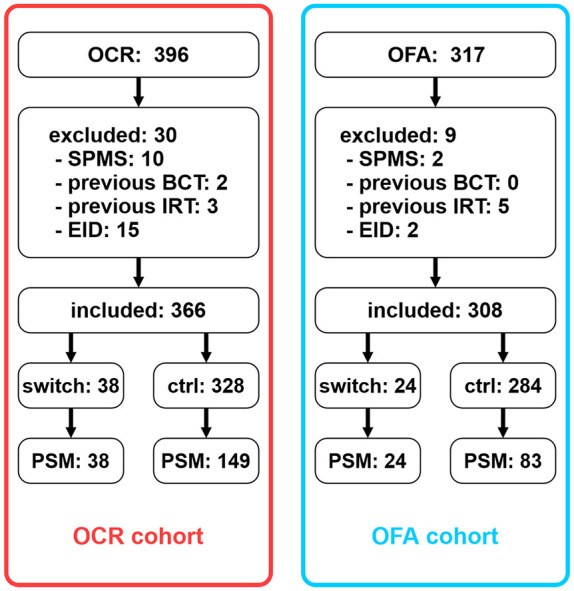
Development of both cohorts that were analyzed in this study. Left column displays the “OCR cohort”; right column displays the “OFA cohort.” All comparisons were made within cohorts. OCR: ocrelizumab; OFA: ofatumumab. SPMS: secondary progressive multiple sclerosis; BCT: B-cell therapy; IRT: immune reconstitution therapy. EID: extended interval dosing. PSM: propensity score matching (here: patients included in the respective cohorts following PSM).

We included 396 patients initiated with OCR between September 2020 and March 2024. Thirty were excluded (Figure 1), leaving 366 patients eligible. Of those, 38 patients underwent a lateral switch to OFA, whereas 328 patients were continuously treated with OCR. Following PSM, 38 switch patients were compared to 149 controls. Mean (±SD) treatment duration was 28.0 ± 10.5 months in the control group and 38.2 ± 7.0 months in the switch group.

Generally, patients were young and early in their disease course, and half of the patients were previously treatment-naïve. Escalation to OCR from platform treatments or fingolimod was observed in about 20% of patients. The remaining patients stopped natalizumab because of JC (John Cunningham) virus antibody seroconversion. These proportions were comparable among switch and control groups. Full baseline demographics are shown in Table 1.

**Table 1.** Baseline demographics of the matched ocrelizumab cohort.

		Switch (38)	Control (149)	SMD
Age at CD20 initiation, years, mean (SD)		*36.3 (8.0)*	*35.1 (9.4)*	*0.0481*
Female patients, No. (%)		*29 (76)*	*114 (76)*	*0.0268*
MS duration since onset, months, mean (SD)		*21.9 (24.3)*	*23.8 (19.9)*	*0.0116*
Relapse rate at baseline, mean (SD)		*0.7 (0.44)*	*0.7 (0.67)*	*-0.0010*
EDSS score at baseline, mean (SD)		*1.8 (0.98)*	*2.0 (1.01)*	***-0.1117***
T2 MRI lesions at baseline, mean (SD)		*17.1 (6.74)*	*17.3 (5.13)*	*0.0105*
Number of previous DMT, mean (SD)		*0.7 (0.86)*	*0.8 (0.96)*	*-0.0071*
Last previous DMT type, No. (%)	treatment-naïve	*20 (53)*	*78 (53)*	*-0.0571*
	beta-interferon	*0 (0)*	*0 (0)*	*-0.0571*
	glatiramer acetate	*3 (8)*	*12 (8)*	*<0.0001*
	dimethyl fumarate	*4 (11)*	*11 (7)*	*0.0244*
	teriflunomide	*2 (5)*	*7 (5)*	*0.0958*
	fingolimod	*2 (5)*	*8 (5)*	*0.0216*
	natalizumab	*7 (19)*	*33 (22)*	*0.0354*

“Switch” refers to patients who were started on ocrelizumab but switched to ofatumumab during follow-up; “control” refers to matched controls who received ocrelizumab throughout. Matching quality is indicated by standardized mean differences between groups. SD: standard deviation; SMD: standardized mean difference; MS: multiple sclerosis; DMT: disease-modifying treatment; EDSS: expanded disability status scale.

**Figure 2.**
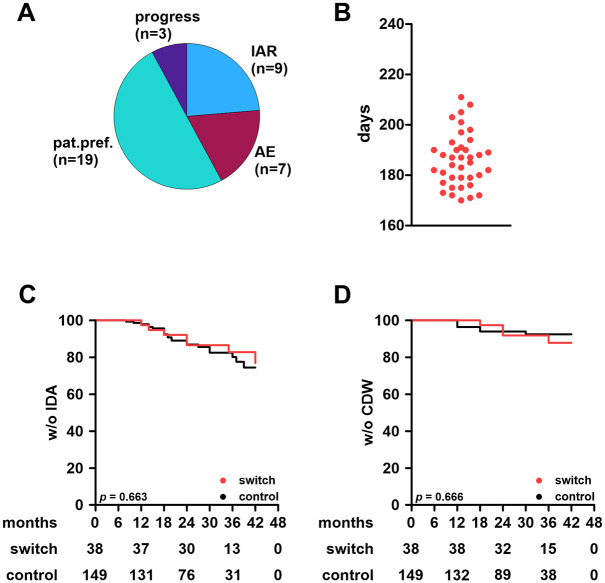
Effectiveness outcomes and conditions of the treatment switch in the ocrelizumab cohort. **A:** Reasons for treatment switch from ocrelizumab to ofatumumab. **B:** time from last dose of ocrelizumab to first dose of ofatumumab among switch patients. **C:** Kaplan-Meier plots for time to first IDA among switch and control patients. Significance was determined using a log-rank test. Numbers at risk are indicated below the plot. **D:** Kaplan-Meier plots for time to first (6 months) CDW. IAR: infusion-associated reaction; AE: adverse event; pat.pref: patient preference; IDA: inflammatory disease activity; CDW: confirmed worsening of disability.

Various reasons for a lateral switch were reported. While half of patients simply switched due to convenience, other patients suffered from infusion-associated reactions upon OCR administration or other adverse events. Notably, only a few patients switched to OFA because of disease activity (Figure 2(a)).

Lateral switches were conducted at the end of the standard dosing interval of OCR (median time from last OCR to first OFA: 186 days (range: 170–211); Figure 2(b)). Since B cells remained depleted in all patients, lateral switches were conducted without saturation phase and patients were directly treated with maintenance dosing.

### OFA cohort

We included 317 OFA patients initiated between September 2021 and March 2024. Nine patients were excluded (Figure 1) and 308 patients were eligible. 24 patients switched from OFA to OCR during follow-up. Following PSM, 24 switch patients were matched to 83 controls. Slight differences within the baseline demographics remained as control patients were slightly older and subsequently had slightly longer disease courses (mean differences of 6 months for both parameters).

Mean (±SD) duration of BCT was 19.3 ± 6.4 months among control patients and 23.7 ± 6.8 months among switch patients. Baseline demographics are shown in Table 2.

**Table 2.** Baseline demographics of the matched ofatumumab cohort.

		Switch (24)	Control (83)	SMD
Age at CD20 initiation, years, mean (SD)		*35.8 (6.8)*	*35.3 (8.9)*	***-0.1805***
Female patients, No. (%)		*17 (71)*	*55 (66)*	*-0.0046*
MS duration since onset, months, mean (SD)		*16.5 (24.7)*	*22.9 (21.3)*	***-0.1303***
Relapse rate at baseline, mean (SD)		*0.9 (0.28)*	*1.0 (0.36)*	*-0.0738*
EDSS score at baseline, mean (SD)		*1.6 (1.06)*	*1.7 (0.88)*	*-0.0201*
T2 MRI lesions at baseline, mean (SD)		*19.5 (6.1)*	*19.0 (6.9)*	*-0.0130*
Number of previous DMT, mean (SD)		*0.4 (0.56)*	*0.4 (0.64)*	-*0.0109*
Last previous DMT type, No. (%)	treatment-naïve	*16 (67)*	*55 (66)*	*-0.0486*
	beta-interferon	*0 (0)*	*0 (0)*	*<0.0001*
	glatiramer acetate	*2 (8)*	*7 (8)*	*-0.0075*
	dimethyl fumarate	*4 (17)*	*12 (15)*	*0.0839*
	teriflunomide	*1 (4)*	*2 (2)*	***0.1251***
	fingolimod	*1 (4)*	*7 (8)*	***-0.1564***
	natalizumab	*0 (0)*	*0 (0)*	*<0.0001*

“Switch” refers to patients who were started on ofatumumab but switched to ocrelizumab during follow-up; “control” refers to matched controls who received ofatumumab throughout. Matching quality is indicated by standardized mean differences between groups. SD: standard deviation; SMD: standardized mean difference; MS: multiple sclerosis; DMT: disease-modifying treatment; EDSS: expanded disability status scale.

**Figure 3.**
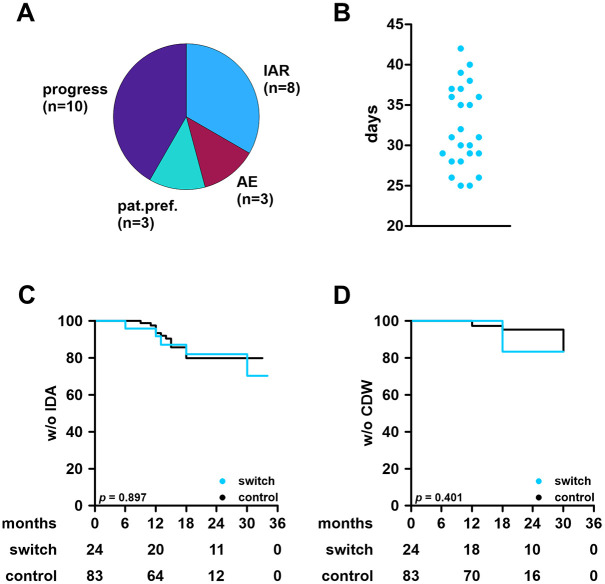
Effectiveness outcomes and conditions of the treatment switch in the ofatumumab cohort. **A:** Reasons for treatment switch from ofatumumab to ocrelizumab. **B:** time from last dose of ofatumumab to first dose of ocrelizumab among switch patients. **C:** Kaplan-Meier plots for time to first IDA among switch and control patients. Significance was determined using a log-rank test. Numbers at risk are indicated below the plot. **D:** Kaplan-Meier plots for time to first (6 months) CDW. IAR: infusion-associated reaction; AE: adverse event; pat.pref: patient preference; IDA: inflammatory disease activity; CDW: confirmed worsening of disability.

Again, the included patients were young and early in their disease course, and two thirds of patients were treatment-naïve, whereas the remaining patients switched from platform treatments or fingolimod. These proportions were well balanced among groups. Predominant reasons for a lateral switch from OFA to OCR were active disease and injection-associated reactions (Figure 3(a)). Lateral switch from OFA to OCR was scheduled at the end of the last standard dosing interval of OFA (median time from last OFA to first OCR: 31 days (range: 25–42); Figure 3(b)). Patients directly received maintenance dosing (600 mg OCR).

### Effectiveness outcomes

During follow-up, 39/187 patients experienced a clinical relapse in the OCR cohort. This corresponds to an annualized relapse rate (ARR) of 0.08 without significant differences between switchers and controls. Among the 7/38 (ARR: 0.06) patients with a relapse in the switch cohort, two relapses were seen prior to switch and five relapses were detected following switch to OFA.

New/enlarging T2L were observed in 38/187 patients (29/149 control patients (19.5%); 9/38 switch patients (23.7%)). Among switchers, four patients developed lesions before the switch and five afterwards. CDW occurred in 20 of 149 control patients (13.4%) and 6 of 38 switch patients (15.8%). Of those six, one developed CDW before and five after switching therapy. Using the Kaplan–Meier method to compare switchers to controls, we found no substantial differences in terms of IDA or CDW (Figure 2(c) and (d)).

In the OFA cohort, we observed clinical relapses in 19/107 patients (ARR: 0.11). These affected 12/83 patients in the control group (ARR: 0.09) and 7/24 in the switch group (ARR: 0.15). Among switch patients, six relapses were observed prior to the switch, only one occurred following switch from OFA to OCR. Numbers are too low to allow the drawing of meaningful conclusions from these slight differences however.

New/enlarging T2L were observed in 23/107 patients (21.4%; control: 15/83 (18.1%), switch: 8/24 (33.3%)). Among switch patients, seven patients developed new/enlarging T2L prior to switch and one patient afterwards.

CDW was observed in 12/107 OFA patients (11.2%), affecting 8/83 control patients (9.6%) and four switch patients (16.7%). Notably, two patients developed CDW 6 months within switch and two patients experienced CDW in their later course. IDA and CDW of both groups were compared using the Kaplan-Meier method and curves did not differ significantly (Figure 3(c) and (d)).

### Development of CD19+ B cells and serum IgG levels

**Figure 4.**
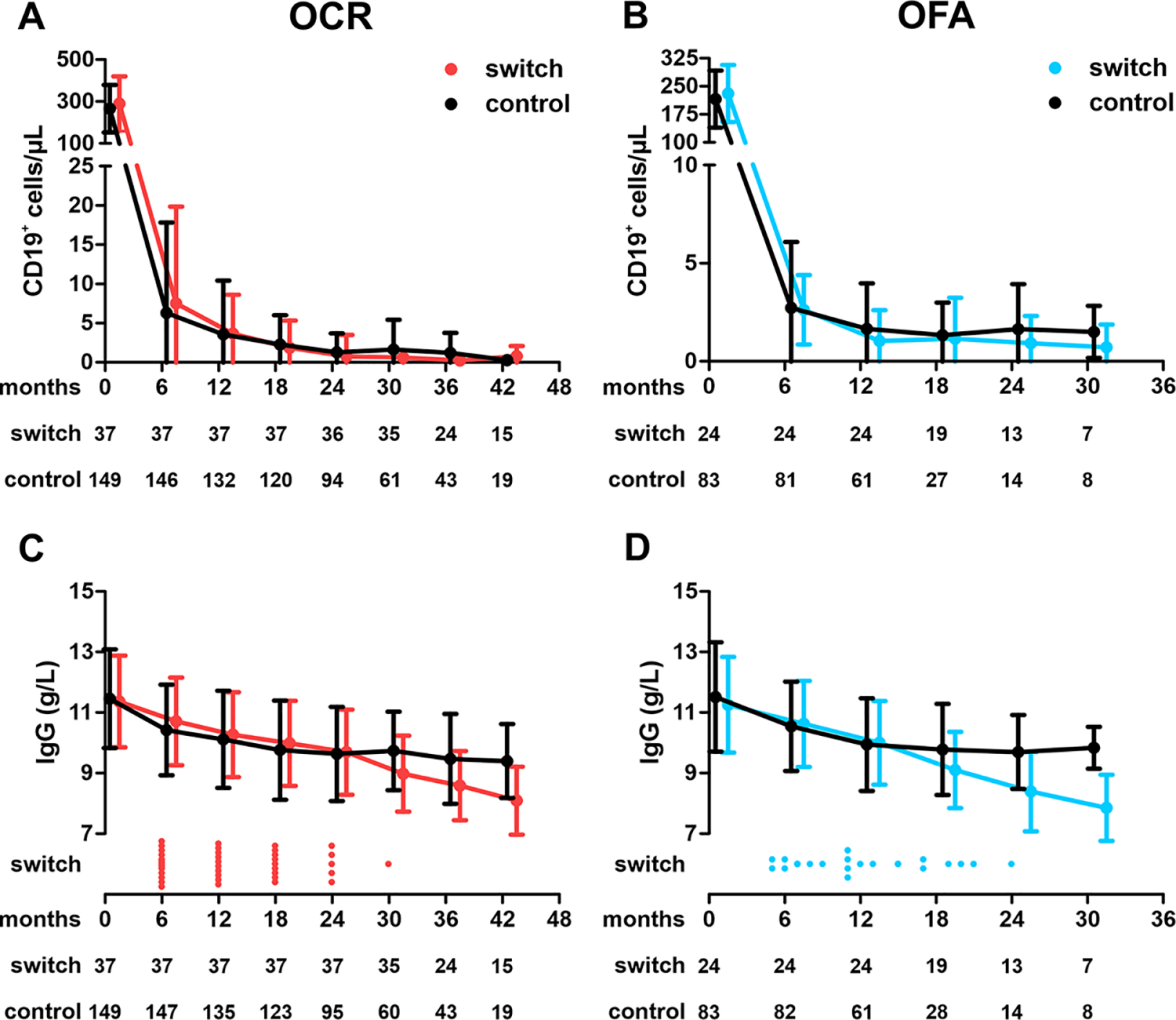
Peripheral CD19+ B cells and serum IgG levels among both cohorts. **A:** Development of peripheral CD19+ B cells among the ocrelizumab cohort. Numbers at risk are indicated below the respective time points. Data are shown as mean ± standard deviation. **B:** Development of peripheral CD19+ B cells among the ofatumumab cohort. **C:** Serum IgG levels among the ocrelizumab cohort. The dot plot below indicates time point of drug switch toward ofatumumab within the switch cohort. Numbers at risk are indicated below the respective time points. **D:** Serum IgG levels among the ofatumumab cohort. OCR: ocrelizumab; OFA: ofatumumab; IgG: Immunoglobulin G.

Among matched OCR patients, we did not observe substantial differences in baseline CD19+ B-cell levels (control: 265.2 ± 113.1/µL; switch: 288.7 ± 130.7/µL). OCR led to a rapid and sustained reduction of peripheral B cells. Of note, B-cell depletion appeared more pronounced in switch patients during the later treatment course. This difference was first visible at month 30 (1.6 ± 3.8/µL (control) vs 0.6 ± 1.0/µL (switch); *p* *=* *0.047*) and persisted at month 36 (*p* *=* *0.025*; Figure 4(a)).

Also in the OFA cohort, baseline levels prior to first BCT were similar (control: 215.8 ± 76.5/µL; switch: 230.7 ± 76.1/µL) and treatment induced rapid and sustained B-cell depletion. In contrast to the OCR cohort, no significant further B-cell reduction was observed after lateral switching from OFA to OCR (month 18: 1.3 ± 1.7/µL vs 1.2 ± 2.1/µL; *p* *=* *0.77*; Figure 4(b)).

We next evaluated the development of serum IgG levels in both cohorts. Baseline levels were comparable (11.5 ± 1.6 g/L (control) vs 11.4 ± 1.5 g/L (switch)). Among the OCR control cohort, we observed the well-described pattern of serum IgG levels characterized by an initial slight decline followed by stabilization. In contrast, switch patients showed a continued decline in IgG levels after switching to OFA. From month 30 on, we saw significant differences in IgG levels (9.7 ± 1.3 g/L vs 9.0 ± 1.3; *p* *=* *0.007*) and findings were consistent in months 36 (*p* *=* *0.010*) and 42 (*p* *=* *0.004*; Figure 4(c)).

Within the OFA cohort, we again found comparable baseline IgG levels (11.5 ± 1.8 g/L vs 11.3 ± 1.6 g/L). Development of serum IgG levels in the control group closely resembled findings made in the OCR control group. Serum IgG levels were also characterized by continued decline following lateral switch to OCR (month 24: 9.7 ± 1.2 g/L vs 8.4 ± 1.3 g/L; *p* *=* *0.016*) and findings were persistent (month 36: *p* *=* *0.003*; Figure 4(d)).

### Adverse events

Adverse events were stratified into “infections,” “other AEs,” “hypogammaglobulinemia” (HGG), and “IARs.” Common infections included urinary and upper-respiratory tract infections, and herpes simplex/zoster; “other AEs” included pruritus, acneiform rash, fatigue, headache, reflux disease as well as menstrual cycle disturbances.

Table 3 summarizes adverse events (AEs) displayed as incidence rates (IR) in the OCR cohort. Infection rates were similar across control and switch groups. “Other AEs” were more frequent in the control group. HGG was more common in the switch group after switching (5/38 had IgG > 7 g/L vs 9/149 in the control group); no cases of severe HGG (<4 g/L) or need for intravenous immunoglobulin (IVIG) substitution occurred. IARs were slightly more often in the switch group before switching (IR 11.45 vs 10.63), but dropped to 2.35 after.

**Table 3.** Incidence of adverse events of the matched ocrelizumab cohort.

OCR	overall (187)	control (149)	switch (38)	
			before switch	after switch
Infections (IR)	*11 (2.35)*	*8 (2.30)*	*2 (2.55)*	*1 (2.35)*
other AE (IR)	*19 (4.05)*	*17 (4.89)*	*2 (2.55)*	*0 (0.00)*
HGG (IR)	*14 (2.99)*	*9 (2.59)*	*0 (0.00)*	*5 (11.76)*
IVIG substitution	*0*	*0*	*0*	*0*
IAR (IR)	*47 (10.02)*	*37(10.63)*	*9 (11.45)*	*1 (2.35)*

“Switch” refers to patients who were started on ocrelizumab but switched to ofatumumab during follow-up; “control” refers to matched controls who received ocrelizumab throughout. IR: incidence rate per 100 patient-years; AE: adverse event; HGG: hypogammaglobulinemia; IVIG: intravenous immunoglobulins; IAR: infusion-associated reaction.

IRs of the OFA cohort for infections, AEs, IARs, and HGG are shown in Table 4. The switch group showed higher IRs for infections, other AEs and IARs than controls, which decreased post-switch. However, absolute numbers are low. HGG occurred in 4/107 patients (2 control, 2 post-switch), without any severe HGG or need to IVIG substitution.

**Table 4.** Incidence of adverse events of the matched ofatumumab cohort.

OFA	overall (107)	control (83)	switch (24)	
			before switch	after switch
Infections (IR)	*8 (4.42)*	*5 (3.75)*	*3 (8.66)*	*1 (4.11)*
other AE (IR)	*9 (4.98)*	*5 (3.75)*	*3 (13.00)*	*1 (4.11)*
HGG (IR)	*4 (2.21)*	*2 (1.50)*	*0 (0.00)*	*2 (8.22)*
IVIG substitution	*0*	*0*	*0*	*0*
IAR (IR)	*23 (12.72)*	*13 (9.74)*	*8 (34.66)*	*2 (8.22)*

“Switch” refers to patients who were started on ofatumumab but switched to ocrelizumab during follow-up; “control” refers to matched controls who received ofatumumab throughout. IR: incidence rate per 100 patient-years; AE: adverse event; HGG: hypogammaglobulinemia; IVIG: intravenous immunoglobulins; IAR: infusion-associated reaction.

## Discussion

Various BCT have entered the therapeutic landscape in MS, all demonstrating superior efficacy and favorable safety in their respective clinical trials. Until now, little is known on effectiveness and especially safety on their sequential use. To address this, we analyzed our prospective, monocentric cohort of patients treated with OFA and OCR. Our study indicates no influence of lateral treatment switches on effectiveness. However, lateral switches were associated with accelerated serum IgG decline. Although our sample size does not permit definitive conclusions, these findings suggest possible interactions between different BCTs that merit further investigation.

Previous data on lateral switch of BCT were published on the switch from rituximab (RTX) to OCR, focussing on infusion-associated reactions. The authors found no substantial decline of serum IgG levels in patients switching from RTX to OCR compared to those who continued RTX in the first year.^7^ To date, only one study has evaluated the switch from OCR to OFA (OLIKOS study).^16^ Unfortunately, this study did not specifically report serum IgG levels. Notable data were derived from OCARINA that had evaluated the switch from OCR iv to OCR sc. Within OCARINA-II, after 12 months serum IgG levels of patients who switched from OCR iv to OCR sc and of patients treated with OCR sc throughout remained comparable.^17^ However, all abovementioned studies were limited by short follow-up periods.

Current evidence indicates comparable effectiveness of OCR and OFA.^4^ In our cohort, we observed a higher proportion of patients switching from OFA to OCR due to disease activity. This might reflect the limited evidence and real-world experience with OFA at the time of switch. Generally, our cohorts presented treatment outcomes closely resembling those reported in pivotal clinical trials.^1,2,5,6^

During long-term follow-up, we observed a reduction of serum IgG levels in patients treated with both OCR and OFA. Whereas the clinical trials of OCR and OFA and their extension studies have shown only minor reductions, real-world data repeatedly demonstrate this phenomenon.^7–9^ Age and treatment duration were clearly associated with an increased risk for development of HGG. Preliminary data indicated that OFA and OCR predispose patients in a comparable manner.^8^ In addition, exposition to previous immunomodulatory or immunosuppressive treatments before BCT was associated with an increased risk for HGG.^8^ We here now observed that also a lateral switch from OCR to OFA and vice versa is associated with accelerated IgG loss compared to continued therapy with one BCT. The underlying mechanism remains unclear and needs further investigation.

Possible contributing factors may include intrinsic properties of the antibodies, such as differences in pharmacokinetics, epitope binding, and mechanism of action, as well as differences in patient characteristics between switchers and non-switchers. While OCR works primarily via antibody-dependent cellular cytotoxicity, OFA mostly induces complement-dependent cytotoxicity.^18^ In addition, data on the impact of both antibodies on B-cell subsets indicate differences in blood and tissue B-cell composition following treatment. Of note, OFA allows a more rapid repopulation of tissue-resident B cells as well as blood-borne plasmablasts in murine models.^19,20^ Moreover, murine studies demonstrated differences in tissue distribution of sc OFA to iv OFA, but not between iv and sc OCR.^21^ These data suggest of a niche-specificity for OFA. We hypothesize that the subsequent administration of OFA and OCR (iv) in the clinical context may result in a more profound depletion of different B-cell compartments. The recent approval of sc OCR could be helpful to delineate whether our findings are predominantly mediated by distinct properties of OCR and OFA or by their route of administration.^17^

Although repeatedly associated with an increased risk for infections in the real-world setting of RTX,^22,23^ extension studies of clinical trials have not shown this in OCR and OFA.^5,6^

Although overall numbers remain low and thus require cautious interpretation, patients in the switch cohort showed higher IRs for infections, other AEs, and IARs. Since these events were among the reasons for switching treatment and IRs declined afterward, we attribute the higher rates to pre-existing conditions prompting the switch, rather than its consequences. Furthermore, rates of infections in the OCR cohort were comparable to previously reported data.^24,25^ Although we observed more cases of HGG among switchers compared to their respective controls, total numbers of cases with manifest HGG remained low. In the OCR group, more patients switched due to adverse events than in the OFA group, yet IgG decline itself was never a reason for switch. However, our study was not specifically designed to address safety concerns. Nonetheless, the overall safety profile remained consistent.

The presented study has some limitations, generally because of its observational design. The number of patients in each switch group was rather low, thus, our results should be considered as “hypothesis-generating” rather than “decision-guiding”. It is of great importance to underline as well that our analyses do not allow any comparisons of the separate cohorts. Furthermore, subgroup analyses between patients with different reasons for switching are not feasible.

Since PSM only accounts for known biases, we cannot rule out the influence of unknown factors, such as an individual higher intrinsic risk for HGG, although the common risk factors (age, treatment duration) were included in our analyses. Overall, our switch patients had slightly longer exposure to BCT compared to controls and thus, we refrained from artificial “re-baselining” and continued to use “first BCT treatment” as baseline to allow unbiased interpretation.

Nevertheless, patients were well-characterized using a standardized follow-up and we achieved well-matched cohorts implementing PSM. In contrast to prior studies, our follow-up duration was substantially longer, adding value to the findings.^24^

To sum up, we provide results of a prospective cohort of young RMS patients who switched their BCT between OCR and OFA compared to respective propensity-score-matched control groups. Clinical effectiveness remained stable following the switch but we observed an association between switching and accelerated IgG decline. Our findings warrant further validation and might help to elucidate niche-specific effects of BCT in RMS.
